# Additive Manufacturing Technologies of High Entropy Alloys (HEA): Review and Prospects

**DOI:** 10.3390/ma16062454

**Published:** 2023-03-19

**Authors:** Tomer Ron, Amnon Shirizly, Eli Aghion

**Affiliations:** Department of Materials Engineering, Ben-Gurion University of the Negev, Beer-Sheva 8410501, Israel

**Keywords:** additive manufacturing (AM), high entropy alloy (HEA), powder bed fusion (PBF), direct energy deposition (DED), binder jetting (BJ), material extrusion (ME)

## Abstract

Additive manufacturing (AM) technologies have gained considerable attention in recent years as an innovative method to produce high entropy alloy (HEA) components. The unique and excellent mechanical and environmental properties of HEAs can be used in various demanding applications, such as the aerospace and automotive industries. This review paper aims to inspect the status and prospects of research and development related to the production of HEAs by AM technologies. Several AM processes can be used to fabricate HEA components, mainly powder bed fusion (PBF), direct energy deposition (DED), material extrusion (ME), and binder jetting (BJ). PBF technologies, such as selective laser melting (SLM) and electron beam melting (EBM), have been widely used to produce HEA components with good dimensional accuracy and surface finish. DED techniques, such as blown powder deposition (BPD) and wire arc AM (WAAM), that have high deposition rates can be used to produce large, custom-made parts with relatively reduced surface finish quality. BJ and ME techniques can be used to produce green bodies that require subsequent sintering to obtain adequate density. The use of AM to produce HEA components provides the ability to make complex shapes and create composite materials with reinforced particles. However, the microstructure and mechanical properties of AM-produced HEAs can be significantly affected by the processing parameters and post-processing heat treatment, but overall, AM technology appears to be a promising approach for producing advanced HEA components with unique properties. This paper reviews the various technologies and associated aspects of AM for HEAs. The concluding remarks highlight the critical effect of the printing parameters in relation to the complex synthesis mechanism of HEA elements that is required to obtain adequate properties. In addition, the importance of using feedstock material in the form of mix elemental powder or wires rather than pre-alloyed substance is also emphasized in order that HEA components can be produced by AM processes at an affordable cost.

## 1. Introduction

**Figure 1:**
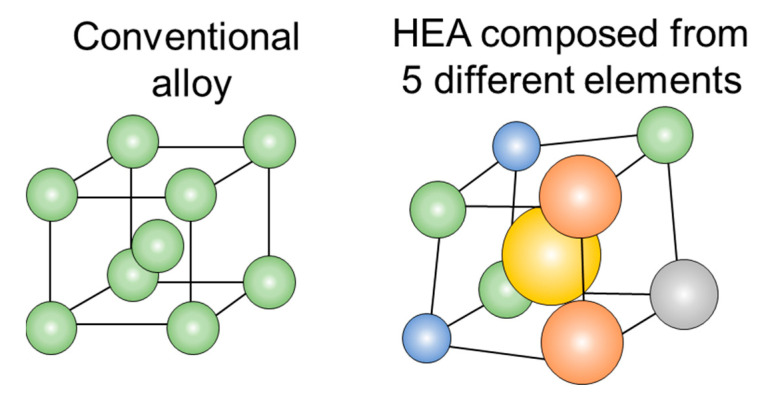
Crystal structure of a conventional alloy vs. high entropy alloys (HEAs).

High entropy alloys (HEAs) offer unique properties, such as high-temperature stability, enhanced specific strength, wear resistance, and excellent corrosion performance in harsh environments. Unlike conventional alloys that typically contain one major element and a small number of alloying elements, HEAs are multi-component systems comprising a few principal elements, as illustrated in Figure 1. They are typically composed of at least five elements, each with individual atomic concentrations ranging from 5−35%. The ability of HEAs to form solid phase solutions is largely due to their configurational entropy and the ability to be synthesized effectively [1,2,3].

The development of HEAs is based on thermodynamic considerations that aim to minimize the Gibbs free energy. This is mainly related to two dominating parameters [4]: (1) the differences in atomic size (δ); and (2) the mix enthalpy ($∆H_{mix}$), as shown by the following equations (for a binary system):

(1) $$\delta =\sqrt{\overset{N}{\underset{i=1,j\neq 1}{\sum }}C_{i}{\left(1-r_{i}/\left(\overset{N}{\underset{i=1}{\sum }}c_{i}r_{i}\right)\right)}^{2}\;}$$

(2) $$\Delta H_{mix}=\overset{N}{\underset{i=1,j\neq 1}{\sum }}4H_{AB}^{mix}c_{i}$$

where $r_{i}$ is the atomic radius of element *i*, $H_{AB}^{mix}$ is the mix enthalpy, and $c_{i}$ and $c_{j}$ are the atomic concentrations of elements *i* and *j*, respectively. It should be pointed out that the main parameter that will determine the stability of the material system is the free energy of the mixed phases ($∆G_{mix}=∆H_{mix}-T∆S_{mix}$). This parameter is closely correlated with the dimension of Ω as defined by the following Equation (3) [4]:

(3) $$\Omega =\frac{T_{m}\cdot \Delta S_{mix}}{\left|\Delta H_{mix}\right|}$$

where $T_{m}$ is the average melting temperature of the elemental ingredients, $∆S_{mix}$ is the mixed entropy, and $∆H_{mix}$ is the mixed enthalpy.

HEAs can be conventionally produced using various methods, such as the arc melting furnace (AMF) [5,6,7], mechanical alloying [8], plasma spark sintering [9], and physical vapor deposition [10]. Lately, there has been considerable interest in producing these alloys by AM technologies, which enable the production of complex components with a minimal amount of post-processing steps.

**Figure 2:**
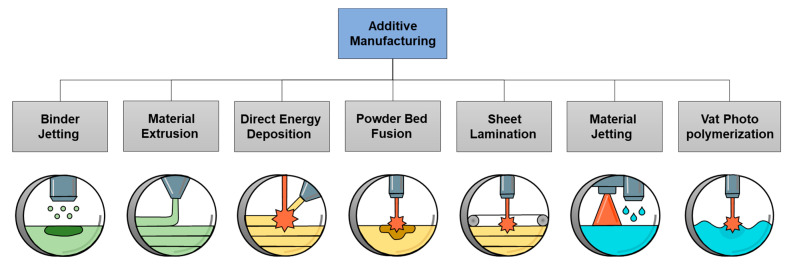
Main types of additive manufacturing (AM) processes according to ASTM F2792—12a.

AM involves building a part layer by layer. As such, most relevant AM technologies commonly use a focused heat source to melt a feedstock material, such as powder or wire, which is then consolidated in subsequent cooling to form a part. AM has gained much attention in recent years due to its unique design capabilities and short lead times. According to ASTM F2792—12a, there are seven main categories of AM processes, as shown in Figure 2. These processes can be divided into three groups: (i) binder-based AM, including binder jetting (BJ) and material extrusion (ME); (ii) high energy heat source AM, including direct energy deposition (DED), powder bed fusion (PBF), and sheet lamination; and (iii) liquid-based AM, including material jetting and vat photopolymerization. Metallic parts can be fabricated using binder-based and high energy heat source technologies.

## 2. Additive Manufacturing (AM) Technologies of High Entropy Alloys (HEAs)

The use of AM technologies to produce HEAs has gained increasing attention in recent years. Various review papers have been published on this topic, focusing on the corrosion performance, powder manufacturing, specific HEA compositions, specific AM technologies and typical microstructures of HEAs produced using AM [11,12,13,14,15,16,17,18,19,20,21]. It is well known that optimizing the processing parameters can improve the mechanical properties of AM-produced HEAs, including refining the microstructure and enhancing properties through post-processing heat treatment [11]. However, synthesizing HEAs such as CrFeCoNi and CrMnFeCoNi HEAs by AM using mixed elemental powder remains challenging due to issues such as elemental segregation and the different melting points of the elements [12]. Imperfections of the AM-produced HEAs such as volume defects along the build direction and elemental segregation can serve as corrosion initiation sites and reduce corrosion resistance. However, controlling the printing parameters can minimize volume defects, and suitable heat treatment can control elemental segregation [19]. The use of selective laser melting (SLM) can improve the microstructure and mechanical strength, but defects such as cracks and porosity may still be present. To date, most research has focused on CrMnFeCoNi and AlCrFeCoNi alloys, but there remains a need for further research on lightweight HEAs [15]. This review aims to provide an overview of the various AM technologies and underline the differences between them in terms of their effects on the microstructure and mechanical properties of HEAs. The rapidly growing number of publications on the topic highlights the need for regular updates on the latest developments in the field.

**Figure 3:**
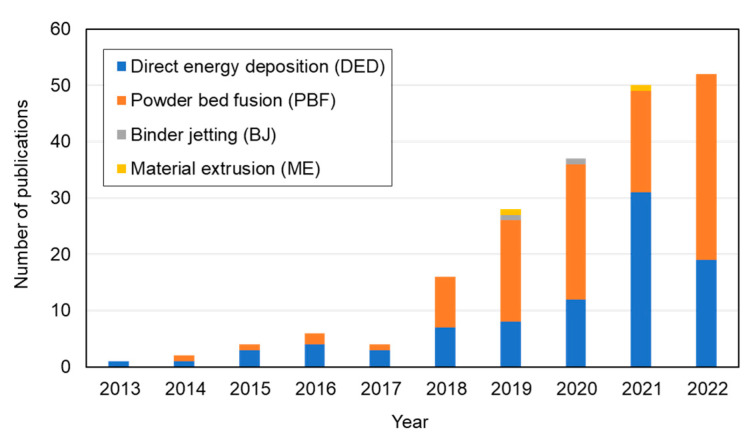
Number of publications on AM of HEAs using direct energy deposition (DED), powder bed fusion (PBF), binder jetting (BJ), and material extrusion (ME) technologies.

The concept of using AM technologies to fabricate HEA components was first introduced by Kunce et al. in 2013 [22]. Since then, an increasingly large number of publications on the AM of HEAs have been published, as shown in Figure 3. This figure is based on the data collected for this review, and clearly indicates the intense interest in using AM to produce HEA components.

Research studies on the AM of HEAs can be broadly classified into four main process technologies: (i) PBF, in which a powder is spread on a printing tray and selectively melted by an energy source, such as a laser beam [23,24,25,26], an electron beam [27], or arc melting [28]; (ii) DED, in which the metal raw material is directly melted layer by layer using different energy sources, such as a laser beam, an electron beam, or arc melting, and the feedstock material can be in the form of powder (using blown powder deposition (BPD) technology [29,30]) or in the form of wire (using arc and laser melting as the heat source—that are usually known as wire arc AM (WAAM) [31,32,33,34] and wire laser AM (WLAM) [35,36]); (iii) BJ, where the powder bed technology uses a liquid binder instead of a heat source, and consequently the printed part is in the form of a green body that requires sintering to obtain adequate density [37,38]; and (iv) ME, where the mixture of powder and binder is extruded through a nozzle to fabricate layer by layer (in this case the printed part is also a green body that requires sintering [39,40]).

**Figure 4:**
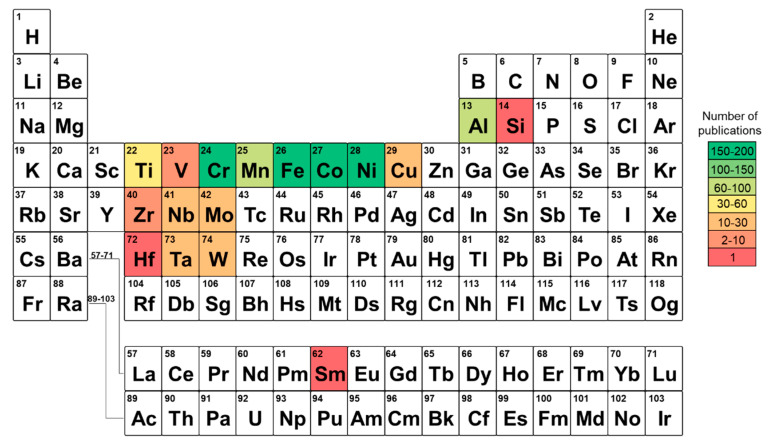
Common alloying elements for HEAs along with number of publications that relate to various combinations of chemical composition.

The selection of alloying elements for HEAs is complex and depends on various thermodynamic factors to obtain desired single phase material, as well as adequate properties and affordable cost. The most common alloying elements are transition metals, such as Cr, Fe, Co, and Ni along with Al, Mn, and Ti, as illustrated in Figure 4. Another group of HEAs produced by AM technologies includes common refractory metals, such as Nb, Mo, Ta, and W.

### 2.1. Raw Materials

**Figure 5:**
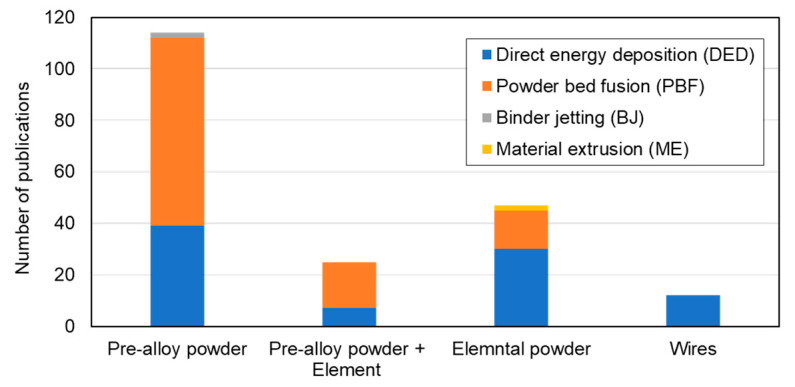
Number of publications related to AM of HEAs using pre-alloyed powder and mix elemental powder by various printing processes.

The synthesis of HEAs involves combining different alloying elements to form a solid solution structure. Conventionally, HEAs are synthesized using an AMF that produces an irregular shape that can be subsequently machined. For most AM technologies, the feedstock material is in the form of powder. In practice, there are two types of powder that can be used. The first type is a pre-alloyed powder, which is synthesized by AMF and produced by gas atomization, hydrogenation, plasma spheroidization, or vacuum electron beam melting [41]. The second type is a mix elemental powder, which is composed of a mixture of the constituent elements. Around 60% of published studies discuss pre-alloyed powder as the feedstock material, as shown in Figure 5. In some research, the authors have modified the composition of a single element to investigate its effect on microstructure formation by a blended powder of pre-alloyed powder with another pure element. In addition, wire can be used as the raw material for DED technology. In this case, pre-alloyed wire can be used, or a combination of three or more commercial wires can be employed.

### 2.2. Powder Bed Fusion (PBF)

**Figure 6:**
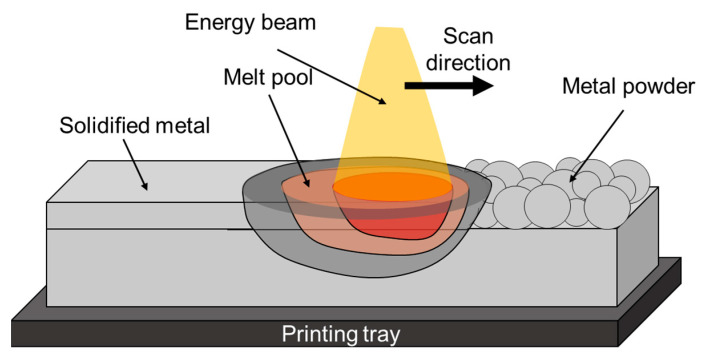
Schematic illustration of typical PBF process.

In PBF technologies, an energy source, such as a laser, an electron beam, or an electric or plasma arc is used to scan each layer of powder. The energy in the beam selectively melts the powder according to the computer-aided design (CAD) model of the part. After one layer has been scanned, the piston of the building chamber moves downward, and another layer of powder is applied. A schematic illustration of the PBF process is shown in Figure 6. An inert atmosphere or vacuum environment is necessary to provide a shielding atmosphere for the molten metal. The main processing parameters in the PBF process are energy/power, scan velocity, layer thickness, and hatch spacing. PBF is known as one of the most accurate AM technologies, allowing the production of complex-geometry parts with a relatively superior surface finish [42,43].

#### 2.2.1. Laser-Based PBF (L-PBF)

PBF, also known as SLM, selective laser sintering (SLS), or direct metal laser sintering (DMLS) [44], is the most common AM method [45]. It uses a laser beam as a power source to selectively melt metallic powder in an inert gas atmosphere, usually argon or nitrogen. L-PBF has the fastest cooling rate of all AM technologies [46], which leads to rapid solidification and fine grain size microstructure that can prevent undesired element segregation, especially in multicomponent systems. However, the rapid cooling rate also creates high residual stress due to the formation of complex dislocation networks during the shrinkage of the melt pool. This can sometimes make it difficult to produce crack-free parts and may necessitate subsequent annealing treatment.

Lin et al. studied the effect of annealing on the mechanical properties of FeCoCrNi HEAs. They found that annealing at 900 °C decomposed the dislocation network, leading to a reduction in residual stress. Higher annealing temperatures of 1100 °C resulted in a further reduction in residual stress and the formation of grains rich in twins through a recrystallization process [47]. Hen et al. discovered that annealing of AlCrFe_2_Ni_2_ led to a reduction in ultimate tensile strength (UTS), while significantly increasing the elongation. The heat treatment caused segregation, leading to the formation of an additional FCC phase. The amount of FCC phase increased with higher heat treatment temperatures [48]. 

The annealing environment also affects the properties of HEAs. Tong et al. investigated the oxidation behavior of CrMnFeCoNi HEAs at high temperatures and found that heat treatment in an ambient atmosphere led to the formation of an oxide layer on the external surface. The thickness of the oxide layer and the irregularity of its morphology increased with temperature [49].

Many studies on the production of HEAs using L-PBF have utilized pre-alloy powders. Those data are presented in Table 1, along with the obtained crystal structure. These powders were commercially produced by a gas atomization process.

Blended powders that consist of pre-alloyed HEA powder mixed with a pure element were also studied in order to evaluate the effect of the additional element on the microstructure and phase composition, as described in Table 2. For example, Sun et al. explored the effect of different Al concentrations (X = 0, 0.1, 0.5, 1) on the microstructure of an Al_X_CrFeCoNi system. They revealed that the Al content was able to change the microstructure from FCC (for X = 0, 0.1) to FCC + BCC/B2 (for X = 0.5) and BCC/B2 (for X = 1) and found that the formation of the dual phase FCC + BCC/B2 in Al_0.5_CrFeCoNi reduced the crack density [72]. Another example is found in the study by Su et al, on the addition of Al (X = 0, 0.5, 0.75, 1) to an Al_X_CrFeNi_2_Cu system. Apparently, the CrFeNi_2_Cu HEA produced by the L-PBF process has a columnar FCC face microstructure that was prone to hot cracks. The addition of Al (X = 0.75) formed a cellular FCC dendrite with a B2 face matrix that reduced crack propagation [102]. 

#### 2.2.2. Electron Beam-Based PBF (EB-PBF)

EB-PBF is a technology that uses an electron beam as the energy source to selectively melt metallic powder. It is also known as electron beam melting (EBM) or selective electron beam melting (SEBM). The electron beam process usually requires a high vacuum environment. Like other PBF technologies, optimization of the printing parameters is necessary to obtain high-density samples. The main printing parameters include electron beam current, scanning speed, layer thickness, and hatch spacing. Before the powder is selectively melted by the electron beam, a pre-heating process (up to 1100 °C) is performed using a defocused electron beam to scan the entire powder surface. This pre-heating process is designed to prevent powder spattering during melting by the electron beam and to reduce residual stresses [110]. However, the pre-heating process is a heat treatment, and the properties of the metal can be affected by the duration of this process. Since the AM process takes time, the lower section of the AM parts may be more affected by this heat treatment.

**Figure 7:**
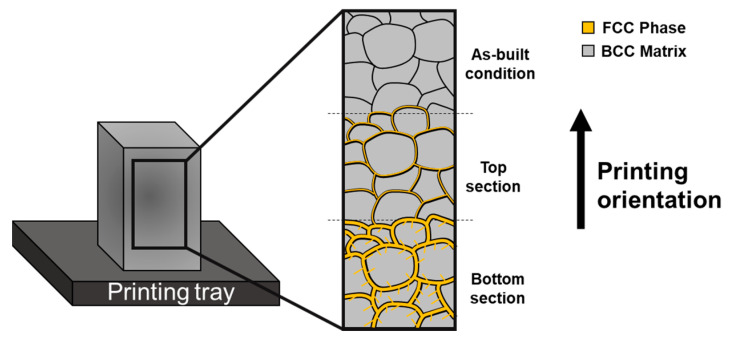
Schematic illustration of the typical microstructure of AlCrFeCoNi and AlFeCoNiCu produced by EB-PBF, showing the formation of FCC phase at grain boundaries due to elemental segregation during the pre-heating process.

The first attempt to use EB-PBF to produce a HEA was performed by Fujieda et al. in 2015 [111]. This was related to an AlCrFeCoNi HEA that was produced by SEBM and resulted in the formation of a two-phase structure. This research was later followed by another 11 studies that also used EB-PBF to manufacture various HEAs, as presented in Table 3. The EB-PBF of AlCrFeCoNi was also studied by Shiratori et al. [112] and Yamanaka et al. [110], with both studies observing different microstructures at the bottom and top sections of the printed parts. The AlCrFeCoNi produced by AMF had a BCC/B2 structure, but the pre-heating process during EB-PBF exposed the part to high temperatures, causing element segregation and the formation of a FCC phase at grain boundaries. As a result, the bottom section, which was exposed to the heat treatment for a longer period, had a higher fraction of the FCC phase than the top part. Similar phenomena were also observed in the preheating of AlFeCoNiCu by Zhang et al. [113], as illustrated schematically in Figure 7.

#### 2.2.3. Mix Elemental Powder as Feedstock Material for PBF Technology

The use of mixed elemental powder in PBF processes is relatively undeveloped, with only 14% of published studies dealing with this approach. This is mainly related to the inherent limitation of using this feedstock material in terms of adequate synthesis of HEAs. However, there are major advantages in using mix elemental powders for manufacturing HEAs. These include more flexibility in composition, lower powder manufacturing costs, and the relatively vast availability of commercial powders [119]. Table 4 presents a list of HEAs that were fabricated using mix elemental powders as feedstock material in PBF processes.

**Figure 8:**
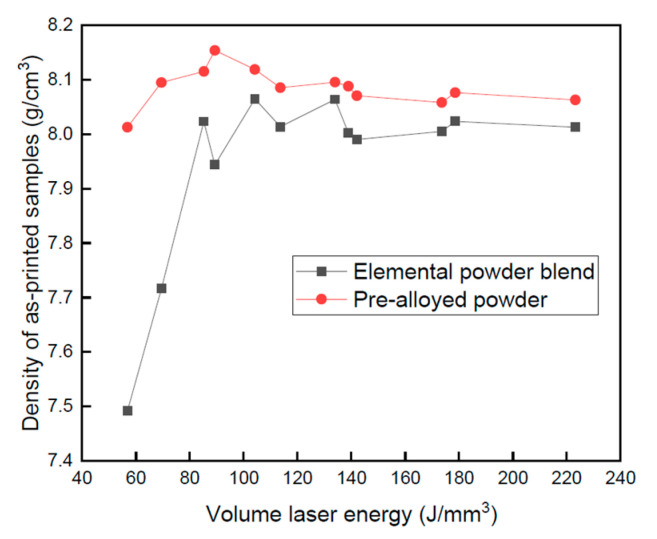
Density of CrFeCoNi HEA using both mix elemental and pre-alloyed powders vs. different volume laser energy by Hou et al. [120].

**Figure 9:**
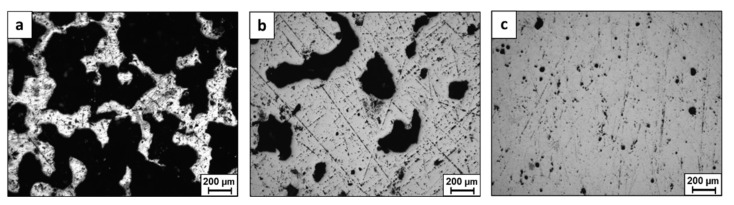
Macroscopic structure by optical microscopy of HEA VNbMoTaW obtained by various LPBF trials using mix elemental powder: (**a**) LPBF—Primary trials, (**b**) LPBF—Intermediate trials, (**c**) LPBF—Advanced trials.

Hou et al. [120] studied the effect of laser energy density on the as-built density of a CrFeCoNi HEA using both mix elemental and pre-alloyed powders, as shown in Figure 8. They found that a higher laser energy density was needed to fabricate the parts from mix elemental powder due to the in situ formation of the HEA. Additionally, they found that under the same printing conditions (apart from the laser power), the density of the pre-alloyed powder samples was higher compared to that of the mix elemental powder samples. Other attempts to produce HEAs using mix elemental powder were carried out by Zhang et al. [121], Huber et al. [122], and Ron et. al. [123] on different alloys having the composition of NbMoTaW and VNbMoTaW. High relative density can be achieved by optimization of the printing parameters. VNbMoTaW HEA produced using L-PBF is shown in Figure 9 and demonstrates an increase from 38% relative density in the initial production attempts to 95% relative density in the advanced attempts. The general insights of those studies were basically in line with those of Hou et al.

**Table 4:** Production of HEAs by PBF process using mix elemental powder as feedstock material.

Alloy	AM System	Crystal Structure	Ref.
Al_0.4_CrFeCoNi	Plasma arc	FCC	[124]
AlCrFeCoNi_2.1_	Electric arc	FCC + BCC	[28]
Al_0.5_CrNbMoTa_0.5_	EB-PBF	BCC + FCC	[125]
Al_0.5_CrNbMoTa_0.5_	EB-PBF	BCC + FCC	[126]
CrFeCoNi	LPBF	FCC	[120,127]
CrMnFeCoNi	LPBF	FCC	[119]
CrMnFeCoNi	Electric arc	FCC	[128]
NbMoTaW	LPBF	BCC	[121]
NiNbMoTa	LPBF	BCC	[129]
Ti_0.5_Ni_0.5_NbMoTa	LPBF	BCC	[129]
TiNbMoTa	LPBF	BCC	[129]
TiVCrFeNi	Electric arc	FCC	[130]
VNbMoTaW	LPBF	BCC	[122,123]

### 2.3. Direct Energy Deposition (DED)

DED is an AM process in which a high energy source, such as a laser beam or plasma arc, is used to melt and deposit material onto a substrate [131]. The feedstock material, which can be either mix elemental powder or pre-alloyed powder, is melted and fused to the previous layer to create metallurgical bonds between layers. The DED process has a higher deposition rate than PBF technologies and is mostly not limited by the size of the printing chamber. However, the surface roughness of DED-produced parts is generally lower and the complexity of parts that can be produced is limited compared to PBF systems [132]. The main processing parameters of the DED process are energy/power, scan velocity, layer thickness, hatch spacing, and material feed rate. These parameters must be carefully controlled to achieve the desired properties.

#### 2.3.1. Blown Powder Deposition (BPD)

**Figure 10:**
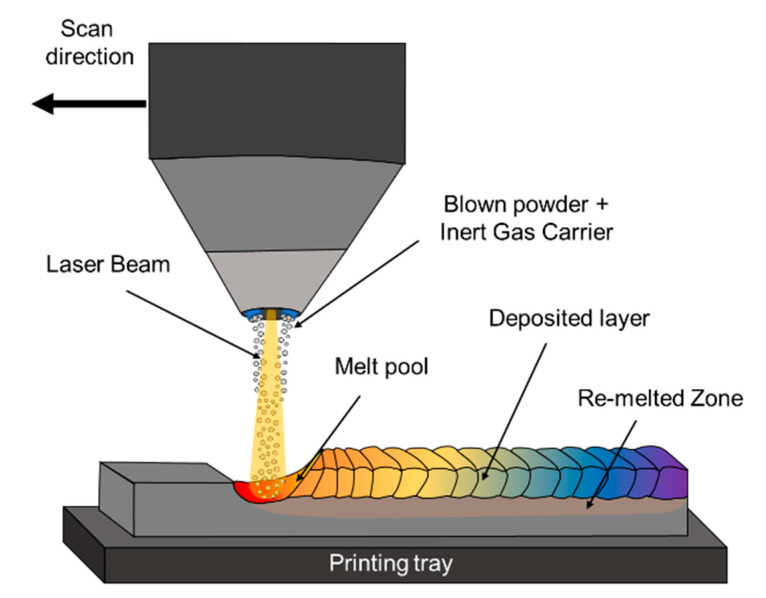
Schematic illustration of blown powder deposition (BFD) process.

Blown powder deposition (BPD) is an AM process that involves directing a laser or plasma beam at metal powder that has been suspended in a high-velocity gas stream. This generates a melt pool from which the part is built up layer by layer, according to a CAD model [133]. BPD is also known as laser metal deposition (LMD), laser powder deposition (LPD), or laser engineered net shape (LENS). A schematic illustration of the BPD process along with the feeding system is shown in Figure 10. A large number of studies using mix elemental powder and pre-alloyed powder to produce HEAs via BPD processes by laser and arc beam energy have been published, as shown in Table 5.

**Figure 11:**
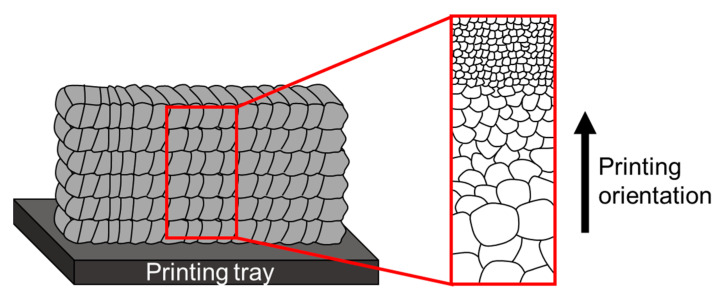
Schematic illustration of the microstructure characteristics as a function of printing orientation of DED-produced parts.

In general, DED technology is known for its high deposition rate, which results in a large number of thermal cycles during the manufacturing process. These thermal cycles can affect the microstructure of the printed parts in various regions. Usually, the top region will retain a fine grain size with high dislocation density, while the bottom region will exhibit a large grain size with low dislocation density. This phenomenon is strongly related to the high number of heat treatment cycles, which is similar to annealing treatment [134]. A schematic illustration of this phenomenon as a function of its printing orientation is shown in Figure 11.

In addition, the heat treatment during the DED process can cause segregation, especially with increased production time. For certain compositions of HEA, such as AlCrFeCoNi, this segregation can lead to significant changes in the microstructure between the bottom and top of the printed part. For example, new FCC phases can be generated in addition to the BCC phase matrix obtained during rapid solidification [135], or alternatively, the fraction of the second phase may increase [136].

The feedstock material used in the DED process, and in particular the type of powder, can also affect the formation of new phases. In the case of a AlCrFeCoNi HEA using pre-alloyed powder [137,138,139], a single solid solution part with BCC/B2 structure is obtained. However, when mix elemental powder is used [140,141,142], the resulting part has a dual FCC + BCC/B2 structure. This can mainly be related to the easier segregation capability in the case of mix elemental powders.

**Table 5:** HEAs produced by BPD process as part of direct metal deposition (DMD) technology.

Alloy	AM System	Feedstock Material	Crystal Structure	Ref.
Al_0.25_FeCoNiCu	Laser	Elemental	FCC	[143]
Al_0.3_CrFeCoNi	Laser	Pre-alloyed	FCC	[144,145,146,147,148]
Al_0.3_CrFeCoNi	Laser	Elemental	FCC	[149]
Al_0.4_CrFeCoNi	Laser	Elemental	FCC	[140]
Al_0.4_CrFeCoNi	Plasma arc	Elemental	FCC	[150]
Al_0.4_CrFeCoNi	Plasma arc	Elemental	FCC + BCC/B2 + σ	[151,152]
Al_0.6_CrFeCoNi	Laser	Elemental	FCC + BCC/B2	[149]
Al_0.6_CrFeCoNi	Laser	Pre-alloyed	FCC + BCC/B2	[148,149]
Al_0.7_CrFeCoNi	Laser	Elemental	FCC + BCC/B2	[140]
Al_0.85_CrFeCoNi	Laser	Pre-alloyed	BCC/B2	[148,149]
Al_0.85_CrFeCoNi	Laser	Elemental	BCC/B2	[149]
AlCrFeCoNi	Laser	Pre-alloyed	BCC/B2	[135,137,138,139]
AlCrFeCoNi	Laser	Elemental	FCC + BCC/B2	[140,141,142]
Al_0.3_CrFeCoNi_1.7_	Laser	Elemental	FCC	[142]
Al_0.7_CrFeCoNi_1.3_	Laser	Elemental	FCC + BCC/B2	[142]
Al_1.7_CrFeCoNi_0.3_	Laser	Elemental	BCC/B2	[142]
Al_0.6_Cr_0.3_Fe_0.3_CoNi	Laser	Elemental	FCC + BCC/B2	[136]
Al_0.6_CrFeCoNi_2.1_	Laser	Pre-alloyed +Al	FCC + BCC/B2	[148,153]
Al_0.7_CrFeCoNi_2.1_	Laser	Pre-alloyed +Al	FCC + BCC/B2	[153]
Al_0.85_CrFeCoNi_2.1_	Laser	Pre-alloyed +Al	BCC/B2	[153]
AlCrFeCoNi_2.1_	Laser	Pre-alloyed +Al	FCC + BCC/B2	[153]
Al_1.1_CrFeCoNi_2.1_	Laser	Pre-alloyed +Al	FCC + BCC/B2	[153]
Al_1.2_CrFeCoNi_2.1_	Plasma arc	Elemental	FCC + BCC/B2	[154]
AlCrFeCoNi_2.1_	Laser	Pre-alloyed	FCC + BCC/B2	[155,156]
AlCrFeCoNi_2.1_	Plasma arc	Elemental	FCC + BCC/B2 + σ	[157]
AlCrFeCoNi_2.1_	Laser	Elemental	FCC + BCC/B2	[158]
AlV_0.3_CrFeMo	Laser	Elemental	BCC	[159]
AlV_7.5_CrFeMo	Laser	Elemental	BCC	[159]
AlV_10_CrFeMo	Laser	Elemental	BCC	[159]
AlV_18.5_CrFeMo	Laser	Elemental	BCC	[159]
Al_2_CrMnFeCoNi	Laser	Pre-alloyed + Al	FCC	[160]
Al_5_CrMnFeCoNi	Laser	Pre-alloyed + Al	FCC + BCC	[160]
Al_8_CrMnFeCoNi	Laser	Pre-alloyed + Al	FCC + BCC	[160]
Al_0.5_FeCoNiCu	Laser	Elemental	FCC	[143]
Al_0.75_FeCoNiCu	Laser	Elemental	FCC + BCC	[143]
AlCrFeNiCu	Laser	Elemental	FCC + BCC	[161]
AlTiCrFeCoNi	Laser	Pre-alloyed	FCC + BCC	[162]
Al_0.3_Ti_0.2_Cr_0.7_FeCoNi_1.7_	Laser	Pre-alloyed	FCC + L1	[163]
AlTiCrFeCoNi	Laser	Pre-alloyed	FCC + BCC + AlNi_3_	[164,165]
AlTiCrFeCoNi	Laser	Elemental	FCC + BCC + AlNi_3_	[166]
AlCrFeCoNiCu	Laser	Pre-alloyed	FCC + BCC	[164,165]
Al_0.17_Ti_0.08_CrFeCoNi	Laser	Pre-alloyed	FCC	[167]
AlCrFeCoNiCu	Laser	Elemental	FCC + BCC	[166]
CrMnFeCoNi	Laser	Pre-alloyed	FCC	[134,160,168,169,170,171,172,173,174,175,176,177,178,179,180,181,182]
CrMnFeCoNi	Laser	Elemental	FCC	[183]
CrFeCoNi	Laser	Pre-alloyed	FCC	[139]
CrFeCoNi	Laser	Pre-alloyed	FCC + µ + σ	[184]
CrFeCoNi	Laser	Elemental	FCC + Co_7_Fe_3_	[185]
CrFeCoNi	Plasma arc	Elemental	FCC	[151,152,186]
CrFeCoNiMo	Laser	Pre-alloyed	FCC	[187]
CrFeCoNiNb	Laser	Elemental	FCC + Laves + MC	[188]
CrFeCoNiNb_0.4_	Plasma arc	Elemental	FCC + Laves	[151,152]
CrFeCoNiTa_0.4_	Plasma arc	Elemental	FCC + Laves	[151,152]
CrFeCoNiW_0.2_	Plasma arc	Elemental	FCC + μ	[186]
CrFeCoNiW_0.5_	Plasma arc	Elemental	FCC + μ	[186]
CrFeCoNiW_0.24_	Plasma arc	Elemental	FCC + BCC + μ	[186]
CrFeCoNiW	Plasma arc	Elemental	FCC + BCC + μ	[186]
CrFeCoNi_0.1_Ta_0.2_	Plasma arc	Elemental	FCC + Fe_7_(NbTa)_3_	[189]
CrFeCoNi_0.3_Ta_0.2_	Plasma arc	Elemental	FCC + Fe_7_(NbTa)_3_	[189]
CrFeCoNi_0.5_Ta_0.2_	Plasma arc	Elemental	FCC + Fe_7_(NbTa)_3_	[189]
CrFeCoNiTa_0.4_	Plasma arc	Elemental	FCC + Co_2_Ta	[189]
Ti_0.6_CrMnFeCoNi	Laser	Pre-alloyed	FCC + BCC	[182]
TiCrMnFeCoNi	Laser	Pre-alloyed	FCC + BCC + σ	[182]
TiCrFeCoNi	Laser	Elemental	FCC + σ + δ + Ni_3_Ti_2_	[190]
TiVCrFeNiZr	Laser	Elemental	C14-Laves + α-Ti	[22]
TiVZrNbMo	Laser	Elemental	BCC+NbTi_4_ + α-Zr	[191]
TiZrNbHfTa	Laser	Elemental	BCC	[192]
TiZrNbTa	Laser	Elemental	BCC	[193]
NbMoTaW	Laser	Elemental	BCC	[194,195,196]
VNbMoTaW	Laser	Elemental	BCC	[197]

#### 2.3.2. Wire Deposition

**Figure 12:**
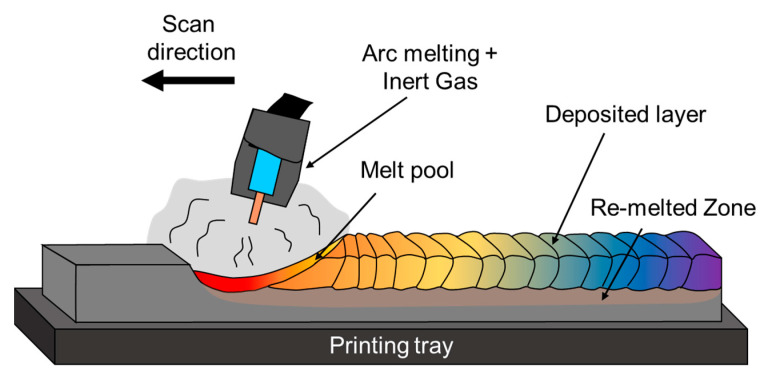
Schematic illustration of wire arc AM (WAAM) process.

Wire deposition, also known as wire arc AM (WAAM), wire laser AM (WLAM), or wire electron beam AM (WEAM), is a DED process that uses welding wire as the raw material. It has gained increased attention in recent years due to its potential for producing large, customized, near-net-shape metal components at a relatively reduced cost. This process involves using TIG welding, MIG welding, plasma torches, laser beams, or electron beams to deposit sequential layers of material from a wire feedstock without the need for extra tooling [198,199]. A schematic illustration of a typical WAAM technology process is shown in Figure 12.

**Figure 13:**
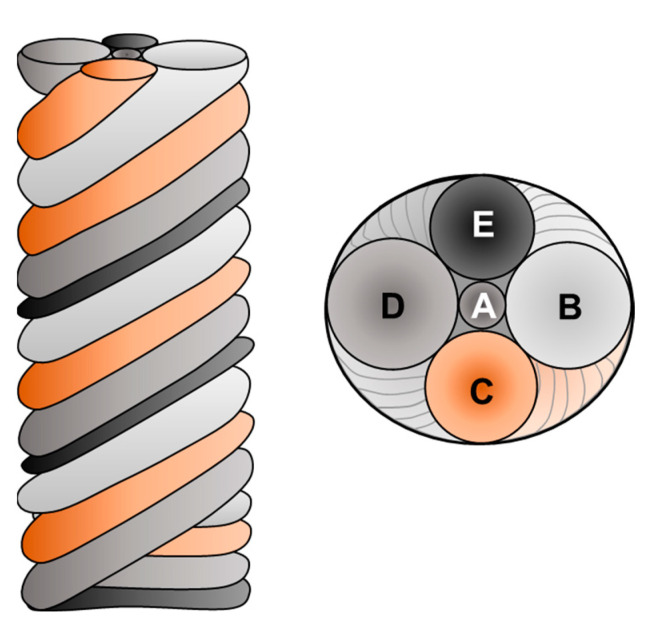
Schematic illustration of CCWs composed with 5 different wires (A–E) for DED process.

**Figure 14:**
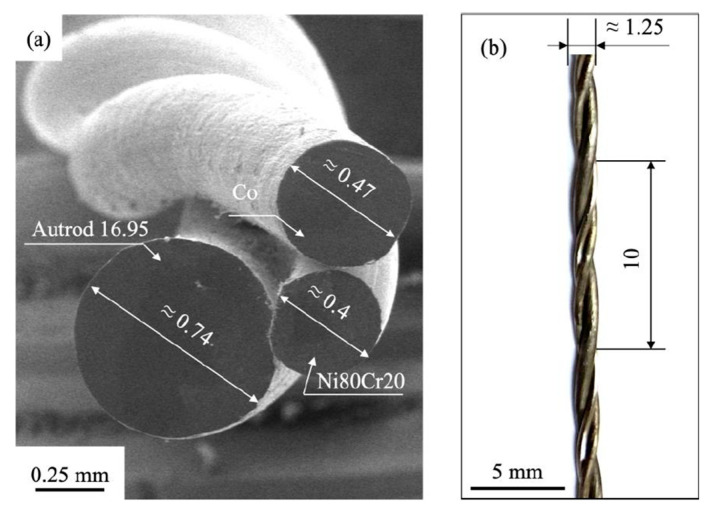
(**a**) SEM image of CCW composed from three stranded wires (pure Co wire, Autrod 16.95 welding wire and Ni80Cr20 wire, (**b**) photograph of the CCW wire [202].

The manufacturing of HEAs using wires as the raw material has been demonstrated by several studies, as shown in Table 6, and has been performed by using a number of commercial welding wires or by fabricating a pre-alloyed welding wire. However, the fabrication of pre-alloyed wires is significantly more expensive than the other options is and quite complex in terms of wire preparation. According to Ahsan et al. [200], the complex process is carried out as follows: (i) melting the HEA elements in an induction furnace; (ii) casting in a cylindrical ingot; (iii) hot isostatic press (HIP) treatment; (iv) machining and extrusion; and (v) wire drawing to construct a 1.2 mm welding wire. To avoid the complexity of fabricating pre-alloyed wires, it is possible to use combined cable wires (CCWs), which consist of 3–7 thin commercial wires, as illustrated in Figure 13. It should be pointed out that the chemical composition of the final HEA depends on the possible combinations of commercial wires; hence, in practice, not every composition is possible. According to Shen et al. [201], the AM HEA produced using wire DED with seven different wires (one Fe wires, two Ni wires, two Al wires, one Co wire and one 304 stainless steel wire) had similar mechanical properties to those of the cast alloy made with the same wires as feedstock material for the AMF process. Other studies [202,203] have demonstrated that the use of three non-pure welding wires can form a single solid solution, as shown in Figure 14.

### 2.4. Binder Jetting (BJ)

**Figure 15:**
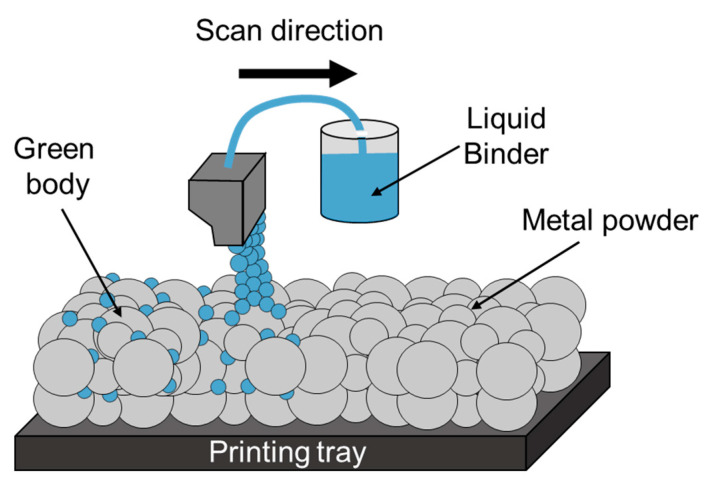
Schematic illustration of BJ process.

BJ is a low-cost AM process that uses a liquid binder to bond powder particles together in order to form a green body that should then be sintered [211]. The powder is spread layer by layer and the binder is applied using an inkjet printing head according to the CAD design. BJ is a unique process that does not use a heat source, and hence the use of pre-alloyed powder as feedstock material is a precondition, as demonstrated by the various studies presented in Table 7. A schematic illustration of the BJ technology process is shown in Figure 15. The main processing parameters in BJ technology relate to the layer thickness, the size and number of the inkjet nozzles, the powder bed temperature, and the printing speed. In addition, the post-processing parameters, which relate to the sintering process, are the sintering temperature and duration.

It has been shown that it is possible to produce HEA components by BJ and sintering; however, there are a few challenges to be addressed. Elevated sintering temperatures reduce the amount of porosity, but also increase shrinkage. In addition, the sintering process, like any other heat treatment, can modify the microstructure and induce the formation of secondary phases. For example, Karlsson et al. found that the sintering of a AlCrFeCoNi HEA at a relatively low temperature (900 °C) resulted in the formation of an undesired sigma phase in addition to the FCC and BCC/B2 phases. In parallel, sintering at high temperatures (above 1200 °C) resulted in the formation of a single BCC phase [212].

### 2.5. Material Extrusion (ME)

**Figure 16:**
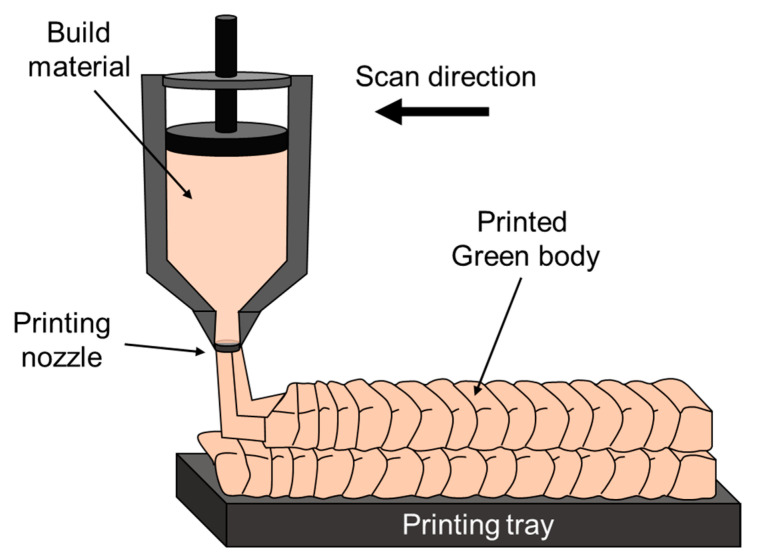
Schematic illustration of ME process using a plunger.

ME is an AM process that uses extrusion to produce components from a soft, malleable, raw material. This process is typically used for producing polymer parts, but it can also be used to produce metallic parts. The feedstock material for metallic parts consists of a mixture of metallic powder and a thermoplastic binder. The feedstock material is fed through a nozzle layer by layer according to a CAD model. There are three options for loading the feedstock material [214]: (i) extruding a rod made from metallic powder and thermoplastic binder using a plunger; (ii) extruding filaments, typically made of polymers; or (iii) extruding a metallic powder and binder using a screw extruder where the rotating screw is able to pump and push the feeding material through a nozzle and toward the printing tray. Following the printing process, a sintering process is required to transform the green body into a high-density metal part. A schematic illustration of the ME process using a plunger is shown in Figure 16. Table 8 introduces a couple of HEAs produced by ME technology using mix elemental powder and pre-alloy as the feedstock material.

### 2.6. Composite Materials

Composite materials with a HEA matrix can easily be created using AM processes that involve powder. These materials can be reinforced with dispersion particles or have a laminated structure. Metal carbides, nitrides, borides, and oxides can be used as reinforcing particles in composite materials with metal alloy matrices [217]. Table 9 introduces different composite materials with a pre-alloyed HEA matrix. An example of a reinforced HEA using TiC nano-ceramic particles demonstrated by Chen et al. [218] showed improved mechanical properties, such as strength and ductility. In this case, the nano-particle reinforcement also enhanced work hardening and grain refinement. Another option for a metal matrix composite is to add refractory elements with a high melting temperature, such as W, as reinforcing particles [219]. BPD systems with two different powder feedstocks can also be used to produce a metal matrix composite by depositing a laminated structure from two different HEAs [220].

### 2.7. Interstitial Doping

Interstitial doping relates to the possibility of adding a small number of interstitial atoms, such as carbon, nitrogen, and oxygen, that can increase the strength of HEAs without decreasing their ductility. For example, the addition of carbon to a FeCoCrNiMn HEA has been shown to increase both strength and ductility [234]. This was due to the modification of the deformation mechanism from relatively pure dislocation glide to combined dislocation glide and twinning. The addition of oxygen to a TiZrHfNb HEA can also enhance strain hardening by creating complexes that promote double cross-slip and the formation of Frank–Read sources that enhance dislocation multiplication [235]. Similarly, the doping of nitrogen into a FeCoNiCr HEA can also increase both strength and ductility by forming low angle boundaries and increasing dislocation networks [236]. Table 10 introduces several examples of HEA systems that incorporate interstitial doping using a L-PBF process and pre-alloyed feedstock material.

### 2.8. Effect of Printing Parameter

**Figure 17:**
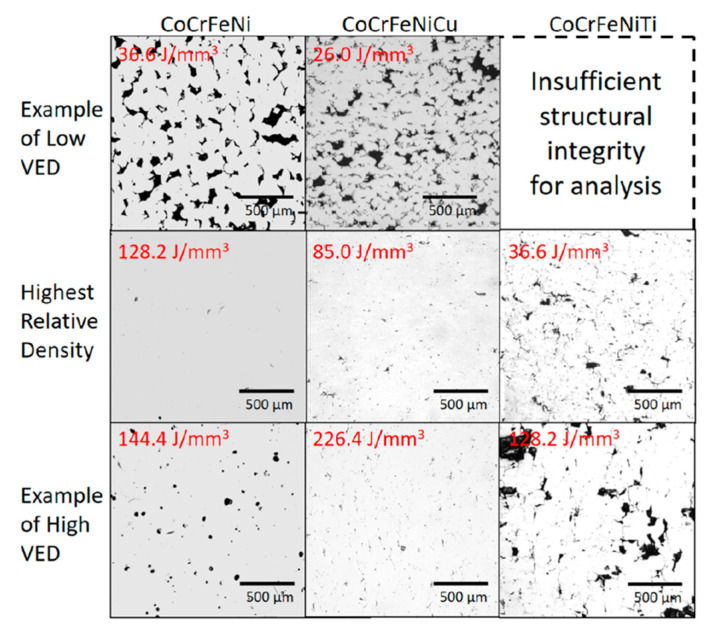
Optical microscopy showing the relative density of three HEA compositions in low, optimal, and high VED (VED values presented in red), Farquhar et al. [101].

To obtain high-density HEA parts of by AM, careful optimization of the printing parameters is required. Inadequate parameters can result in the production of inherent defects. For example, excessively high energy density can cause over-melting and vaporization within the melt pool, while insufficient energy density can lead to incomplete fusion of melt pools. Table 11 presents the printing parameters used for some common HEA compositions in various AM processes. In general, increasing the energy density increases the density until reaching optimal printing parameters, beyond which the relative density may decrease due to increased defects from vaporization and splashing of the melt pool. However, increasing the energy density can improve surface finish [75] and increase the lattice parameter [173]. The applied energy density also affects the microstructure; for example, relatively low energy density can result in cellular microstructures, while high energy density can produce lamellar structures due to the higher cooling rates [56]. The microstructure of HEA produced by the DED process can be affected by the printing time, where the lower section of the part undergoes more thermal cycles. Xiang et al. [172] showed that the printing parameters can affect this phenomenon. For example, relatively low energy density can result in a columnar structure in the lower and middle sections, with a mixed columnar and equiaxed grain structure in the top part. In parallel, relatively high energy density can result in an equiaxed grain structure throughout the alloy. The optimal energy density depends on the feedstock material used. The effect of volume energy density (VED) on relative density has been investigated by Farquhar et al. [101], as shown in Figure 17. CrFeCoNi pre-alloyed powder was mixed with Cu powder and Ti powder, and the optimal energy density varied due to differences in the melting points between the CrFeCoNi (1414 °C), Cu (1084 °C), and Ti (1688 °C) powders. In addition, using elemental substances with lower melting points can affect the microstructure. At relatively high energy density, vaporization of an element reduces its amount, and this can lead to significant microstructural modifications.

### 2.9. Overall Comparison between AM Processes

A comparison of various AM processes used to produce HEAs in terms of production characteristics and microstructural phenomenon—PBF, DED, BJ and ME—is shown in Table 12. This mainly relates to feedstock material characteristics, printing design and parameters, and the related microstructures that are obtained. Altogether, it is clear that while PBF and DED processes tend to produce microstructures with preferred orientations, and consequently preferred properties, BJ and ME mostly produce a relatively large equiaxed grain structure. In addition, BJ and ME processes always required a post sintering phase, while PNF and DED do not need special post-processing treatment (apart from stress relief in the use of PBF when the printing is carried out on a cool tray).

## 3. Mechanical Properties

**Figure 18:**
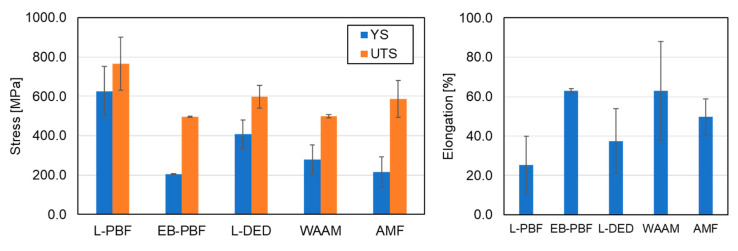
The effect of different AM technologies on tensile strength of CrMnFeCoNi HEA in terms of YS, UTS, and elongation. L-PBF [45,74,75,82,84,87,203], EB-PBF [115], L-DED [160,168,170,171,172,173,174,180], WAAM [202], AMF [87,172,246].

It is apparent that AM process parameters have a significant effect on the microstructure, and consequently, on the mechanical properties of HEAs. This insight relates to the well-known AM technologies used to produce HEA components. The most studied HEA composition is CrMnFeCoNi, which has been the focus of many publications. This was mainly attributed to the fact that this HEA does not form new phases during element segregation. The mechanical properties of CrMnFeCoNi HEAs produced by various AM processes in as-built condition are displayed in Figure 18, which reveals that the highest yield strength (YS) and UTS were obtained by the L-PBF process. The relatively increased strength can be attributed to the very high cooling rate of the L-PBF process, which leads to high residual stress and relatively reduced elongation. A satisfactory balance between the strength and ductility is usually obtained by an annealing treatment that is able to reduce strength and increase elongation.

The CrMnFeCoNi HEA produced by DED using BPD with a laser as the energy source showed relatively high strength with increased elongation compared to the case of L-PBF. Here, the DED process undergoes a high number of thermal cycles, which acts as an annealing heat treatment that leads to stress relief. The HEAs produced by EB-PBF and WAAM processes showed relatively similar mechanical properties. Overall, it seems that the differences in mechanical properties using the same energy source are minor and can be modified by post-processing heat treatment. In addition, the mechanical properties obtained by L-DED were similar to the HEA produced by conventional AMF process. 

**Figure 19:**
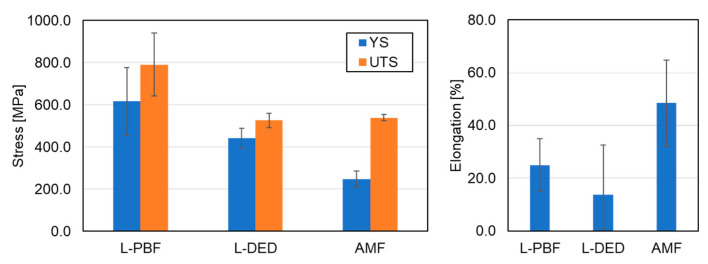
The effect of different AM technologies on tensile strength of Al_0.3_CrFeCoNi HEA in terms of YS, UTS and elongation. L-PBF [53,105], L-DED [145,146], AMF [247,248].

An additional example of the mechanical properties of Al_0.3_CrFeCoNi HEA produced by L-PBF and L-DED processes in comparison to the AMF process is shown in Figure 19. The L-PBF process demonstrated an increase in strength compared to both L-DED and AMF processes.

## 4. Corrosion Performance of HEAs Produced by AM Processes

HEAs are known for their superior corrosion resistance in extreme environments. However, their corrosion performance depends on the microstructure and inherent defects produced during the AM process. According to Niu et al. [52], the corrosion resistance of AlCrFeCoNi HEAs improves due to an increase in energy density. Finer microstructures with a large amount of B2 phase are obtained at high energy densities, which enhances the corrosion resistance. Higher energy density also reduces the number of defects (such as pores and lack of fusion) that can serve as initiation sites for pitting corrosion [161]. According to Kuwabara et al. [114], the use of EB-PBF for producing AlCrFeCoNi HEAs can cause element segregation due to preheating, leading to the formation of pitting sites in the Cr-depleted areas, whereas in the cast samples, pitting forms only at the grain boundaries. Another artifact of EB-PBF is microstructural changes in different sections of the printed material. Yamanaka et al. [110] found that the corrosion resistance in the bottom parts of AlCrFeCoNi HEAs (rich with FCC phases) is better than that in the top parts (with no FCC phases). Furthermore, the corrosion resistance of CrMnFeCoNi HEA produced by the LPBF process with single phase microstructure was improved compared to the cast samples due to relative grain refinement [61]. In parallel, VNbMoTaW HEAs showed similar corrosion resistance in LPBF and the cast samples, with a reduction in the pit dimensions compared to the cast samples [123].

## 5. Concluding 

HEAs have earned significant attention in recent years due to their unique properties in terms of mechanical properties, thermal stability, and corrosion performance in hostile environments. However, the conventional methods for producing HEAs, such as AMF, mechanical alloying, plasma sintering, and physical vapor deposition, are extremely limited due to the complexity of the production process and consequent high cost. Hence, the potential of producing HEA components by AM, which are able to fabricate complex, near-net-shape components at an affordable cost, is quite attractive. Currently, the status of the most important AM technologies that are used to produce HEA parts, such as PBF, DED, ME and BJ, is well into the development phase. 

Overall, the major limitation of all the main AM processes concerns the critical effect of the printing parameters on the properties of the final product. This limitation relates to two important aspects: (i) adequate synthesis between the elements composing the HEA to produce a homogeneous crystal structure with suitable microstructure and phase composition; and (ii) adequate physical properties, mainly with regard to printing defects and density in as-printed conditions and following post-processing heat treatment. 

Relating to the feedstock material in the form of powder or wires that are used by the PBF and DED processes, it is preferable to work with a mixed elemental substance rather than a pre-alloyed material, especially if low cost is sought. In the case of mixed elemental powder, proper selection of the different powders in terms of grain size and morphology is essential. In the case of CCWs for DED processes, the wire assembly and cross section size should comply with the characteristics and capabilities of the energy source. This requires proper selection of different wire diameters and careful consideration of their plastic forming capabilities. In all cases, the criterion for success should focus on adequate synthesis and physical properties in terms of microstructural defects.

## Figures and Tables

**Table 1:** HEAs fabricated by L-PBF process and pre-alloyed powders as feedstock material.

Alloy	Crystal Structure	Ref.
AlCrFeCoNi	BCC/B2	[50,51,52]
Al_0.3_CrFeCoNi	FCC	[53]
Al_0.5_CrFeCoNi	FCC	[54]
AlCrFeCoNi_2.1_	FCC	[55]
AlCrFeCoNi_2.1_	FCC + BCC/B2	[56,57,58,59]
AlCrFe_2_Ni_2_	BCC/B2	[48]
Al_0.55_Cr_0.5_FeNi	BCC/B2	[60]
AlCrFeNiCu	BCC/B2	[61]
AlCrFeNi_2_Cu	FCC + BCC/B2	[62]
AlCrFeNi_2.5_Cu	FCC + BCC/B2	[62]
AlCrFeNi_2.75_Cu	FCC + BCC/B2	[62]
AlCrFeNi_3_Cu	FCC + BCC/B2	[62]
Al_0.3_CrFeCoNiCu	FCC	[63]
AlFeCoNiCu	FCC + BCC/B2	[64]
CrFeCo_1.1_Ni_1.6_	FCC + BCC	[65]
CrFeCoNi	FCC	[47,66,67,68,69,70,71,72,73]
CrMnFeCoNi	FCC	[45,46,49,74,75,76,77,78,79,80,81,82,83,84,85,86,87]
TiMoTaW	BCC + HCP	[88]
Ti_2.3_Zr_2.3_NbMoTa	BCC	[89]
Al_2_CrFeCo_3_Ni_3_Mo_0.1_W_0.1_	FCC + BCC/B2	[90]
AlCrFeCoNiCu	FCC + BCC/B2	[91]
Al_0.2_Ti_0.3_CrFeCo_1.5_Ni_1.5_	FCC	[92]
Al_0.3_Ti_0.23_FeCoNi	FCC	[93]
Al_0.25_Ti_0.25_FeCoNi	FCC	[94]
Al_0.5_V_0.2_Cr_0.9_FeNi_2.5_	FCC	[95]
Si_0.25_Cr_0.75_MnFe_2_Co	FCC + HCP	[96]
Si_0.25_Cr_0.75_MnFe_2_CoCu_2_	FCC + HCP	[97]
Si_0.25_Cr_0.75_MnFe_2_Co	FCC + HCP	[98]
Ti_0.5_CrFeCo_1.5_Ni_1.5_Mo_0.1_	FCC + HCP	[99]
CrFeCoNi	FCC	[100]
CrFeCoNi	FCC	[101]
CrFeNi_2_Cu	FCC	[102]
TiCr_2.5_FeCoNi_2_W_0.5_	BCC	[103]
TiCr_4_Fe_9_Ni_6_W	FCC	[104]

**Table 2:** LPBF production of HEAs using a blend of pre-alloyed and pure element powders as feedstock material.

Alloy	Feedstock Material	Crystal Structure	Ref.
AlCrFeCoNi	Pre-alloyed + Al	BCC/B2	[72]
Al_0.01_CrFeCoNi	Pre-alloyed + Al	FCC	[73]
Al_0.05_CrFeCoNi	Pre-alloyed + Al	FCC	[73]
Al_0.1_CrFeCoNi	Pre-alloyed + Al	FCC	[72,73]
Al_0.3_CrFeCoNi	Pre-alloyed + Al	FCC + BCC/B2	[105]
Al_0.5_CrFeCoNi	Pre-alloyed + Al	FCC + BCC/B2	[72]
Al_0.5_CrFeNi_2_Cu	Pre-alloyed + Al	FCC + BCC/B2	[102]
Al_0.75_CrFeNi_2_Cu	Pre-alloyed + Al	FCC + BCC/B2	[102]
AlCrFeNi_2_Cu	Pre-alloyed + Al	FCC + BCC/B2	[102]
AlCrFeCoNi_2.1_	Pre-alloyed + pre-alloyed	FCC + BCC/B2	[106]
AlCrFe_2.3_Ni_2.3_	Pre-alloyed + Fe + Ni	FCC + BCC/B2	[107]
CrMnFeCoNi	Pre-alloyed + Mn	FCC	[108]
CrMnFeCoNi	Pre-alloyed + pre-alloyed	FCC	[109]
CrFeCoNiCu	Pre-alloyed + Cu	FCC	[101]
Ti_0.2_CrFeCoNi	Pre-alloyed + Ti	FCC	[100]
Ti_0.4_CrFeCoNi	Pre-alloyed + Ti	FCC	[100]
Ti_0.6_CrFeCoNi	Pre-alloyed + Ti	FCC	[100]
TiCrFeCoNi	Pre-alloyed + Ti	FCC + HCP + NiTi	[101]

**Table 3:** HEAs fabricated by EB-PBF process using pre-alloyed powders as feedstock material.

Alloy	Crystal Structure	Ref.
AlCrFeCoNi	FCC + BCC/B2	[110,111,112,114]
AlFeCoNiCu	FCC + BCC/B2	[113]
CrMnFeCoNi	FCC	[115,116,117]
CrMnFeCoNiTi_0.18_	FCC + σ + γ + D0_24_	[117]
CrMnFeCoNiTi_0.5_	BCC + σ + γ + D0_24_	[117]
CrMnFeCoNiTi_2_	BCC + σ + γ + D0_24_	[117]
CrFeCoNiMo	FCC + SC + Ni_3_Ti	[118]

**Table 6:** Fabrication of HEAs by DED process using wire as feedstock material.

Alloy	AM System	Feedstock Material	Crystal Structure	Ref.
Al_0.1_CrFeCoNi	Arc welding	Pre-alloyed Wire	FCC	[200]
AlCrFeCoNi	Arc welding	7 Wires	FCC + BCC/B2	[201,204,205]
Al_0.7_Cr_0.4_FeCo_0.34_Ni_2_	Electron beam	3 Wires	SC	[206,207]
Al_2.1_Cr_0.5_FeCo_0.3_Ni_2.1_	Arc welding	3 Wires	BCC/B2	[208]
CrMnFeCoNi	Arc welding	3 Wires	FCC	[202]
CrMnFeCoNi	Electron beam	3 Wires	FCC	[203]
SiCrMnFeCoNi	Arc welding	3 Wires	FCC	[209]
TiNbMoTaW	Arc welding	7 Wires	BCC	[210]

**Table 7:** HEAs fabricated by BJ process and pre-alloyed powders.

Alloy	Sintering Temperature	Crystal Structure	Ref.
AlCrFeCoNi	900 °C	FCC + BCC/B2 + σ	[212]
AlCrFeCoNi	1000 °C, 1100 °C	FCC + BCC/B2	[212]
AlCrFeCoNi	1200 °C, 1300 °C	BCC/B2	[212]
CrMnFeCoNi	1150 °C, 1250 °C	FCC	[213]

**Table 8:** HEAs fabricated by ME process.

Alloy	Binder	Feedstock Material	Crystal Structure	Ref.
CrFeCoNi	PLGA + DBP	Elemental	FCC	[215]
CrMnFeCoNi	THF + 2-Butoxyethanol + PMMA-PnBA	Pre-alloyed	FCC	[216]

**Table 9:** Composite materials with HEA matrix.

Alloy	Type	AM	Raw Material	Crystal Structure	Ref.
CrMnFeCoNi	Reinforcing particles	L-PBF	Pre-alloyed + TiN	FCC + TiN	[221,222]
CrMnFeCoNi	Reinforcing particles	L-PBF	Pre-alloyed + nano TiC	FCC	[218,223]
CrMnFeCoNi	Reinforcing particles	L-DED	Pre-alloyed + TiC	FCC + TiC	[224,225]
CrMnFeCoNi	Reinforcing particles	L-DED	Pre-alloyed + WC	FCC+M_23_C_6_	[225,226]
CrMnFeCoNi	Reinforcing particles	L-DED	Pre-alloyed + TiB_2_	FCC + TiB_2_	[227]
CrMnFeCoNi	Reinforcing particles	L-DED	Pre-alloyed + W	FCC + BCC + Fe_7_W_6_	[228]
CrFeCoNi	Reinforcing particles	PA-DED	Elemental + SiC	FCC+Cr_7_C_3_	[229]
CrFeCoNi	Reinforcing particles	L-PBF	Pre-alloyed + W	FCC + W	[219]
SiCrMnFeCo	Reinforcing particles	L-PBF	Pre-alloyed + B_4_C	FCC+HCP	[230]
NbMoTaW	Reinforcing particles	L-PBF	Pre-alloyed + W + WC	BCC + NbC	[231]
CrMnFeCoNi + 304 SS	Laminated structure	L-DED	Pre-alloyed + 304SS	FCC	[232]
AlCrFeCoNi + CrFeCoNi	Laminated structure	L-DED	Pre-alloyed	FCC/BCC	[233]
AlTi_0.5_CrFeCoNi + CrMnFeCoNi	Laminated structure	L-DED	Pre-alloyed	FCC/BCC	[220]

**Table 10:** Interstitial doping of HEAs fabricated by L-PBF process and pre-alloyed feedstock material.

Alloy	Interstitial Doping	Crystal Structure	Ref.
CrFeCoNi	N	FCC	[236,237]
CrFeCoNi	C	FCC	[238,239,240]
CrMnFeCo	C	FCC + HCP	[241]
CrMnFeCoNi	C	FCC	[242,243]
CrMnFeCoNi	N	FCC	[244,245]

**Table 11:** Printing parameter of selected HEA compositions produced by different AM processes.

Alloy	AM System	Feedstock Material	Powder Size [µm]	Printing Parameters	Ref.
Al_0.1_CrFeCoNi	WAAM	Pre-alloyed Wire	X	Current: 200 A Voltage: 15.5 V Travel speed: 3.3, 10 mm/s Feed rate: 2000 mm/min Energy density: 82.23, 120 J/mm^3^	[200]
Al_0.3_CrFeCoNi	L-DED	Pre-alloyed	74–150 Average: 110	Power: 1000 w Travel speed: 1000 mm/s Layer thickness: 30 μm	[145]
Al_0.3_CrFeCoNi	L-PBF	Pre-alloyed	20–42 Average: 29	Power: 150–170 w Scan speed: 1100–1300 mm/s Layer thickness: 25–30 μm Hatch spacing: 45 μm Energy density: 85–137 J/mm^3^	[53]
AlCrFeCoNi	L-PBF	Pre-alloyed	3.8–53 Average: 28.6	Power: 250–400 w Scan speed: 1000 mm/s Layer thickness: 40 μm Hatch spacing: 90 μm Energy density: 68.4–111 J/mm^3^	[52]
AlCrFeCoNi	EB-PBF	Pre-alloyed	45–105	Beam Current: 4.5–9 mA Preheating temperature: 950 °C Scan speed: 215 mm/s Layer thickness: 70 μm	[112]
AlCrFeCoNi	L-DED	Pre-alloyed	75–150	Power: 950 w Travel speed: 4 mm/s Feed rate: 9.5 g/min Layer thickness: 30 μm	[135]
AlCrFeCoNi	L-DED	Elemental	50–150	Power: 800 w Travel speed: 13 mm/s Layer thickness: 25 μm	[141]
AlCrFeCoNi	WAAM	7 Wires	X	Current: 156 A Voltage: 16.2 V Travel speed: 8–12 mm/s Feed rate: 5.5 m/min	[201]
AlCrFeCoNi_2.1_	L-PBF	Pre-alloyed + Ni	10–75 Average: 32.7	Power: 200–400 w Scan speed: 600–1000 mm/s Energy density: 88.9–190 J/mm^3^	[56]
CrFeCoNi	L-PBF	Elemental	19-73	Power: 100–250 w Scan speed: 700–1100 mm/s Layer thickness: 20 μm Hatch spacing: 80 μm Energy density: 57–223 J/mm^3^	[120]
CrFeCoNi	L-PBF	Pre-alloyed	18–49 Average: 30.1	Power: 94 w Scan speed: 582 mm/s Layer thickness: 30 μm Hatch spacing: 42 μm Energy density: 128.2 J/mm^3^	[101]
CrMnFeCoNi	L-PBF	Pre-alloyed	5–45 Average: 36	Power: 400 w Scan speed: 800–4000 mm/s Layer thickness: 40 μm Hatch spacing: 90 μm Energy density: 37–185 J/mm^3^	[75]
CrMnFeCoNi	L-PBF	Pre-alloyed	20–80 Average: 43.4	Power: 150 w scan speed: 600 mm/s layer thickness: 60 μm hatch spacing: 50 μm Energy density: 83 J/mm^3^	[79]
CrMnFeCoNi	EB-PBF	Pre-alloyed	44.1–88.7 Average: 65	Current: 2–14 mA Preheating temperature: 900–980 °C scan speed: 492–3446 mm/s layer thickness: 50–70 μm hatch spacing: 50–70 μm	[115]
CrMnFeCoNi	L-DED	Pre-alloyed	Average: 120	Power: 1000–1400 w Travel speed: 13 mm/s feed rate: 7–9 g/min	[172]
CrMnFeCoNi	L-DED	Pre-alloyed	10–90	Power: 600–1000 w Travel speed: 13 mm/s feed rate: 7–9 g/min	[173]

**Table 12:** Comparison of production characteristics and microstructure phenomena of different AM processes used to produce HEAs.

Production Characteristics and Microstructure Phenomenon	PBF	DED	BJ	ME
Feedstock material	Powders Pre-alloyed and elemental	Powders and wires Pre-alloyed and elemental	Powder Pre-alloyed	Powder paste Pre-alloyed
Powder size	Few microns	No limitation	Few microns	No limitation
Heat source	Laser beam Electron beam Arc melting	Laser beam Electron beam Arc melting	No heat source	No heat source
Deposition rate	Low	High	High	High
Post-processing requirements	Stress relief	No requirements	Sintering	Sintering
Printing geometry	Complex	Simple	Complex	Simple
Accuracy	High	Low	High	Low
Multi-material Production ability	One material	Multi-material ability	One material	Multi-material ability
Typical microstructure	Preferred orientation (columnar /dendrite structure) For EB-PBF increase in grain size in lower section of the part	Preferred orientation (columnar /dendrite structure) Increase in grain size in lower section of the part	Relatively large equiaxed grain structure	Relatively large equiaxed grain structure

## Data Availability

No new data were created or analyzed in this study. Data sharing is not applicable to this article.
